# Sepsis-Induced Endothelial Barrier Dysfunction: Mechanisms, Pathology, and Therapeutic Advances

**DOI:** 10.34133/research.0997

**Published:** 2025-11-21

**Authors:** Rui Wang, Qiuju Han, Jiangbo Fan, Zhe Xu, Wenyi Liu, Di Liu, You Li, Juan Du, Jianhui Sun, Huacai Zhang, Qingli Cai, Chu Gao, Jianxin Jiang, Zhen Wang, Ling Zeng

**Affiliations:** ^1^ State Key Laboratory of Trauma and Chemical Poisoning, Daping Hospital, Army Medical University, Chongqing 400042, China.; ^2^ Department of Intensive Care Unit, Daping Hospital, State Key Laboratory of Trauma and Chemical Poisoning, Army Medical University, Chongqing 400042, China.; ^3^ Department of Emergency, the Affiliated Hospital of Guizhou Medical University, Guizhou Medical University, Guiyang 550001, China.

## Abstract

Sepsis is a life-threatening disease characterized by systemic inflammation and endothelial barrier dysfunction, leading to multiorgan failure and high mortality. This review provides a comprehensive overview of the pathological mechanisms underlying sepsis-induced endothelial dysfunction, focusing on glycocalyx degradation, endothelial cell death, increased vascular permeability, and coagulopathy. During sepsis, the endothelial glycocalyx (EG) is disrupted, leading to increased vascular permeability and impaired microcirculation. Endothelial cells undergo various forms of cell death, including apoptosis, pyroptosis, ferroptosis, and autophagy, which are driven by inflammation and oxidative stress. These processes are further complicated by the activation of coagulation pathways and the formation of intravascular thrombi. The interaction between endothelial cells and immune cells amplifies the inflammatory response, contributing to the persistence of systemic inflammation. We also discuss emerging therapeutic strategies aimed at protecting endothelial cells, modulating inflammation, and improving coagulation function, including glycocalyx protectants, anti-inflammatory agents, anticoagulants, and endothelial repair mechanisms. Future research should focus on translating these therapeutic approaches into clinical practice to improve outcomes in patients with sepsis.

## Introduction

Sepsis is a systemic inflammatory response syndrome (SIRS) caused by infection that leads to multiorgan dysfunction and even failure. Common areas of infection include the lungs, abdomen, and urinary system. Sepsis-induced multiorgan dysfunction poses a severe threat to the lives of patients. The mortality rate of sepsis ranges from 10% to 20%, whereas that of septic shock is even higher, at approximately 40% to 50% [1]. SIRS-induced endothelial barrier disruption is a main feature of sepsis. Morphological and functional changes in endothelial cells, which constitute a barrier between tissues and the bloodstream, occur during the regulation of barrier permeability, coagulation, and inflammatory responses caused by endotoxins and cytokines [2,3]. Endothelial dysfunction is a key pathological event during sepsis, as it leads to vascular leakage, uncontrollable inflammation, and abnormal coagulation/thrombosis, resulting in multiple organ failure and mortality [4]. During sepsis, pathogenic microorganisms can activate endothelial cells through exogenous pathogen-associated molecular patterns (PAMPs) and endogenous damage-associated molecular patterns (DAMPs) while also causing structural damage to endothelial cells, which leads to increased vascular permeability [5]. The activation of pattern recognition receptors, including Toll-like receptors (TLRs), prompts endothelial cells to be reprogrammed into a proinflammatory phenotype. Activated endothelial cells can participate in the disease process of sepsis through the release of cytokines, chemokines, and procoagulant factors and through the expression of adhesion molecules. Glycocalyx damage and endothelial cell apoptosis lead to increased vascular barrier permeability, resulting in tissue edema [6]. Studies have shown that appropriate sepsis-related endothelial changes can limit bacterial spread while assisting in the recruitment of white blood cells to clear bacteria. However, persistent and severe sepsis-related changes in endothelial cell phenotypes impair microcirculatory blood flow, cause tissue hypoperfusion, and lead to multiorgan failure.

To reduce the mortality rate of sepsis, exploring the pathophysiological mechanisms of endothelial cell and endothelial barrier changes during sepsis and identifying potential therapeutic approaches are essential. Therefore, this review focuses on the mechanisms underlying the interaction between endothelial cells and macrophages, aiming to provide new insights into and perspectives for the pathophysiology and treatment of sepsis.

## Pathological Injury to Endothelial Cells and the Endothelial Barrier in Sepsis

### Damage to the glycocalyx

The endothelial glycocalyx (EG) is a layer of glycosaminoglycans and proteoglycans rich in heparan sulfate that covers all healthy vascular endothelia and constitutes the innermost structure of the vasculature [6]. Proteoglycans and glycosaminoglycans are the main components of the EG. Highly hydrated glycosaminoglycans form a dense gel-like layer on the surface of the glycocalyx, and their negative charge endows the glycocalyx with a high reflection coefficient for albumin. The EG can also attach to circulating plasma proteins to form the endothelial surface layer (ESL), which is crucial for maintaining vascular barrier function and organ perfusion [7]. Consequently, the glycocalyx is highly important for maintaining vascular integrity and enabling leukocyte transport. With the advancement of in vivo imaging techniques, the phenotypic changes in the glycocalyx during sepsis have been better validated. Tang et al. [8] conducted nanomechanical analyses of microvessels via atomic force microscopy and reported that the thickness of the EG decreases after stimulation with lipopolysaccharide (LPS), tumor necrosis factor-α (TNF-α), and other agents. Three-dimensional ultrastructural visualization via electron microscopy has been used to observe changes in the glycocalyx of capillaries in the heart, kidneys, lungs, and other organs before and after sepsis [9]. Molema et al. [10] reported that glycocalyx disruption can lead to increased permeability to large molecules and increased leukocyte adhesion in the renal cortical microcirculation. During sepsis, the EG in pulmonary vessels rapidly sheds, and endothelial heparanase is activated by TNF-α, leading to the shedding of heparan sulfate from the EG. Adhesion molecules such as E-selectin and ICAM-1 previously harbored in the EG are exposed and activated and cause granulocyte adhesion and a profound inflammatory response [11]. Shed components of the glycocalyx, such as syndecan-1 and hyaluronan, can be detected in the plasma of patients with sepsis [12]. The barrier function of pulmonary vascular EG can be restored by plasma products, a process that is critically dependent on the expression of syndecan-1. In the absence of syndecan-1 expression, plasma products have no protective effect on pulmonary EG [13].

However, endothelial cells exhibit substantial functional heterogeneity depending on the vascular bed and tissue in which they reside [14,15]. This functional heterogeneity is crucial for understanding the diverse pathological mechanisms underlying sepsis-induced endothelial dysfunction. For example, the disruption of the EG during sepsis is not uniform across different tissues. In the lungs, the glycocalyx is particularly susceptible to degradation by neutrophil elastase and metalloproteinases, leading to significant vascular leakage [11]. In contrast, in the brain, tight junctions are more abundant, providing a stricter barrier that is relatively more resistant to sepsis-induced damage [16]. However, even in the brain, sepsis can lead to localized disruptions in the blood–brain barrier, contributing to neuroinflammation [17].

### Endothelial cell death

Cell death, also termed programmed death, is a mechanism through which organisms regulate homeostasis. Under physiological conditions, endothelial cells achieve self-regulation by inducing the death of a small proportion of cells [3]. During sepsis, the number of dying endothelial cells significantly increases. Endothelial cells can undergo several types of cell death, including apoptosis, pyroptosis, ferroptosis, and autophagy. The main factors leading to endothelial cell death during sepsis are inflammation and oxidative stress [5]. The hypoxia-inducible factor-1α (HIF-1α) signaling pathway can be used by PD-L1 to trigger the inflammatory response in pulmonary endothelial cells, resulting in increased endothelial cell apoptosis [18]. LPS can increase the phosphorylation of YAP at Y357 in human pulmonary microvascular endothelial cells (HPECs). This phosphorylation causes YAP to translocate from the cytoplasm to the nucleus, where it binds to the transcription factor P73 and triggers apoptosis in endothelial cells [19]. In LPS-induced acute kidney injury, activated endothelial calpain promotes the phosphorylation of p38, leading to increased expression of inducible nitric oxide synthase (iNOS); this results in the excess production of nitric oxide (NO) and reactive oxygen species (ROS), which cause endothelial cell (EC) apoptosis [20]. Pyroptosis is an inflammatory form of cell death that is commonly associated with septic shock and tissue injury. The main difference between pyroptosis and apoptosis lies in the disruption of cell membrane integrity. The contents released from cells amplify local inflammatory responses, leading to immune imbalance and exacerbated tissue damage. The 3 main mechanisms of endothelial pyroptosis in sepsis include the GSDMD-NT (gasdermin D N-terminal domain)-dependent pathway, the NLRP3 (nucleotide-binding domain, leucine-rich-containing family, pyrin domain-containing-3)-dependent pathway, and intercellular communication. These mechanisms involve multiple molecular and intercellular interactions, collectively leading to endothelial cell pyroptosis. The NLRP3 inflammasome is instrumental in driving sepsis-induced pyroptosis in endothelial cells [21]. In endothelial cells, extracellular cold inducible RNA binding protein (eCIRP) can activate the NLRP3 inflammasome via the TLR4/MD2- and nuclear factor κB (NF-κB)-dependent pathways, increasing the expression of intercellular adhesion molecule 1 (ICAM1) and activating the inflammatory response. This activation subsequently induces the expression of caspase-1, interleukin-1β (IL-1β), and IL-18, ultimately leading to endothelial cell pyroptosis [22]. During sepsis, oxidative stress reactions in endothelial cells are exacerbated, leading to the accumulation of lipid peroxidation products, which ultimately induce ferroptosis in endothelial cells [23]. Autophagy, a form of regulated cell death (RCD), plays a dual role in sepsis: It helps clear microbes and regulate inflammatory responses and leads to cellular dysfunction and exacerbated inflammation when excessively activated. Consequently, during advanced stages of infection, sustained autophagy activation can trigger uncontrollable vascular leakage and overactive inflammation [24].

### Increased vascular permeability caused by vascular dystonia and endothelial barrier breakdown

A monolayer of endothelial cells forms the vascular endothelium and plays crucial roles in vascular integrity, regulation, barrier function, the inflammatory response, and coagulation. The intercellular junctions between endothelial cells are equally important for these functions. Complex endothelial junctional structures, including gap junctions and adherens junctions, form the endothelial barrier, which is vital for maintaining vascular integrity and enabling communication between endothelial cells and surrounding tissues [25]. Adherens junction proteins [such as vascular endothelial (VE)-cadherin] are crucial for inhibiting endothelial cell growth upon contact and regulating the paracellular permeability of circulating leukocytes and solutes. Tight junction proteins (such as occludin and claudins) mainly serve as barriers in the cell membrane to regulate paracellular permeability and preserve cell polarity. These junction proteins typically exist in complex with intracellular scaffold proteins such as ZO proteins. Gap junctions (formed by proteins such as connexin) are communication structures that allow small molecules and solutes to pass between adjacent cells. The arrangement and expression levels of endothelial junctional complexes are influenced by the specific blood vessel type and the permeability demands of the perfused organ. Tight junctions are abundant in endothelial junctions in the brain, ensuring strict control of blood–brain barrier permeability [16]. In contrast, postcapillary venules have fewer tight junctions, making them more prone to inflammation and immune cell extravasation [26].

Endothelial cells synthesize NO, which is essential for maintaining vascular tone. The generation of NO is modulated by shear stress in blood vessels and signaling molecules such as vascular endothelial growth factor (VEGF) and adenosine. The dysregulation of NO activity is a primary cause of vascular tone dysregulation in patients with sepsis. However, abrogating NO dysregulation alone does not improve vascular tone in these patients [27]. Endothelial cells also produce prostacyclin, which promotes vasodilation and prevents platelet deposition on the vascular wall. Endothelial cells can produce potent vasoconstrictors such as endothelin-1 and contribute to the conversion of angiotensin-1 into the potent vasoconstrictor angiotensin-2. Both play important roles in maintaining vascular tone [28,29]. Furthermore, ferroptotic endothelial cells can induce the formation of neutrophil extracellular traps (NETs) through the release of HMGB1, thereby exacerbating vascular inflammation and compromising the vascular barrier [30].

In sepsis, neutrophil elastase and metalloproteinases degrade the extracellular domain of VE-cadherin, leading to vascular barrier disruption [31]. ADAM10 is a membrane-anchored metalloproteinase that participates in intercellular adhesion and inflammatory signaling. During sepsis, endothelial ADAM10 is activated; it cleaves VE-cadherin, disrupts interendothelial junctions, and increases vascular permeability [32]. Studies have identified endothelial ADAM10 as a key nodal molecule through which certain bacteria (e.g., *Staphylococcus aureus* and *Pseudomonas aeruginosa*) precipitate microvascular injury in sepsis: Its activation dismantles the endothelial barrier, triggers von Willebrand factor (VWF) release and platelet aggregation, and ultimately precipitates organ dysfunction [33]. ADAM17, known as TNF-α converting enzyme (TACE), is a pivotal enzyme in inflammatory responses and mediates the shedding and release of multiple inflammatory mediators, such as TNF-α, IL-6R, and VCAM-1. Studies in sepsis animal models have shown that reducing ADAM17 expression decreases serum levels of IL-1β, IL-6, and TNF-α while also decreasing ICAM-1 and VCAM-1 expression in lung and liver tissues, thereby significantly improving 72-h survival in mice [34]. In sepsis, the TNF-α/NF-κB pathway—an essential signaling axis that drives endothelial inflammation—is activated by ADAM17, thereby inducing endothelial inflammation, apoptosis, and barrier dysfunction [35]. VE-cadherin junctions are strictly regulated by Rho proteins, with the activation of the key subtype Rac1 and the inhibition of RhoA being crucial for stabilizing the VE-cadherin complex and preventing vascular leakage [36]. Moreover, cytokines such as IL-1β and TNF-α induce the tyrosine phosphorylation of VE-cadherin, leading to the disruption of endothelial adherens junctions and subsequent endothelial barrier dysfunction. Atorvastatin enhances vascular stability by inhibiting ANGPT2 release and reducing VE-cadherin phosphorylation [37]. The disruption of key endothelial adhesion molecules is mediated by key proinflammatory cytokines in sepsis. For example, TNF-α disrupts endothelial tight junctions through an NF-κB-dependent mechanism, particularly affecting claudin-5 [17,27]. During sepsis, tissue hypoxia and inflammatory responses stimulate the expression of VEGF, which promotes angiogenesis to improve tissue oxygenation. However, elevation of VEGF expression also increases vascular permeability, leading to the leakage of fluid and proteins from the vasculature into the interstitial space and ultimately causing tissue edema and organ dysfunction [38]. VEGF can disrupt the structures of tight junctions and adherens junctions between endothelial cells, increasing intercellular gaps [39]. VEGF has been reported to induce the reorganization of the actin cytoskeleton, leading to endothelial cell contraction and intercellular gap formation, causing fluid leakage [40].

The complement system is a critical component of innate immunity and orchestrates immune surveillance and tissue homeostasis. Its excessive activation or dysregulation can trigger chronic inflammation and tissue damage, thereby driving the pathogenesis of multiple diseases. Complement system activation leads to the formation of the membrane attack complex (MAC), which can cause endothelial cell damage [41]. Additionally, uncontrolled complement activation is a key pathogenic driver in sepsis: Large amounts of anaphylatoxins such as C3a and C5a are released, activating immune cells (e.g., neutrophils and macrophages) and inducing a cytokine storm (e.g., TNF-α and IL-6) together with a thromboinflammatory response. These events further exacerbate endothelial dysfunction, increase endothelial barrier permeability, promote microthrombus formation, and ultimately precipitate multiple organ dysfunction syndrome (MODS) [42].

## Increased Endothelial Barrier Permeability and Sepsis-Associated Coagulopathy

Under normal physiological conditions, endothelial cells are essential for maintaining vascular homeostasis. Endothelial cells maintain the fluidity of blood within blood vessels through their anticoagulant properties, promoting a favorable environment for anticoagulant proteins such as tissue factor (TF) pathway inhibitors, protein C, and antithrombin. Moreover, endothelial cells resist thrombosis by synthesizing and expressing heparan sulfate proteoglycans, key elements of the glycocalyx that bind and enhance the function of plasma anticoagulant proteins [43]. Additionally, under physiological conditions, fibrinogen and fibronectin contribute to endothelial integrity by interacting with the EG and integrin receptors (e.g., αvβ3 and α5β1). Fibrinogen reinforces adherens junction stability by linking VE-cadherin to the cytoskeleton, whereas fibronectin serves as an extracellular matrix scaffold that regulates endothelial cell spreading and barrier permeability [44,45]. Fibronectin is deposited onto the subendothelium before platelets arrive after vascular injury; it can cross-link with fibrin to increase thrombus stability, but in the absence of fibrin, it paradoxically inhibits platelet aggregation, exerting a self-limiting regulatory effect. As the first responders to hemostasis, platelets interact with fibrinogen and fibronectin through integrins such as αIIbβ3, thereby promoting thrombus formation and achieving effective hemostasis [46,47]. Moreover, resting platelets continuously release platelet-derived extracellular vesicles (pEVs) that are rich in sphingosine-1-phosphate (S1P), transforming growth factor-β (TGF-β), and angiopoietin-1, all of which play essential roles in maintaining vascular endothelial barrier integrity [48].

**Fig. 1.**
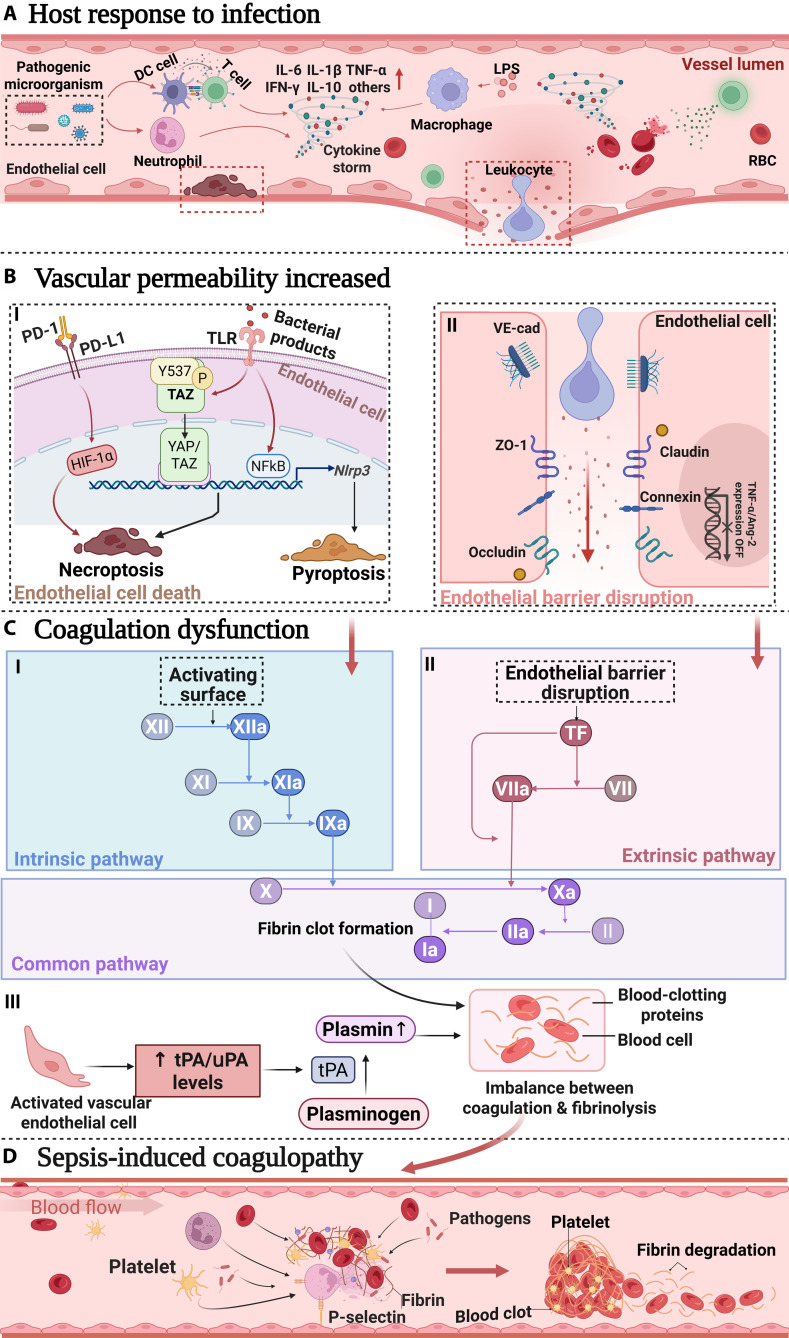
Schematic diagram of sepsis-induced endothelial cell death and increased endothelial permeability causing coagulopathy. (A) Host response to infection: During sepsis, pathogenic microorganisms activate dendritic cells (DCs), T cells, and neutrophils, triggering a cytokine storm that releases cytokines such as IL-6, IL-1β, TNF-α, interferon-γ (IFN-γ), and IL-10. Macrophages and leukocytes are also activated and interact with endothelial cells. (B) Increased vascular permeability. (I) Endothelial cells recognize bacterial products through PD-1/PD-L1 and TLR receptors, activating the NF-κB signaling pathway, which leads to NF-κB translocation into the nucleus, causing endothelial cell necrosis and apoptosis. (II) Endothelial cells maintain barrier function through junction proteins such as ZO-1, VE-cadherin, and claudin. The presence of LPS and cytokine storms leads to the down-regulation of the expression of these junction proteins, disrupting the endothelial barrier. (C) Coagulation dysfunction: Damage to endothelial cells activates coagulation pathways, including (I) the intrinsic pathway (through factors XII, XI, IX, and X and fibrin clot formation) and (II) the extrinsic pathway (through TF, VIIa, Xa, and fibrin clot formation). (III) Activated vascular endothelial cells release tissue-type plasminogen activator/urokinase-type plasminogen activator (tPA/uPA), increasing tPA/uPA levels, promoting fibrin degradation, and disrupting the balance between coagulation and fibrinolysis. (D) Sepsis-induced coagulopathy pathway: Blood flow involves pathogens, platelets, and fibrin, leading to platelet activation and P-selectin expression. This process results in the formation of blood clots and subsequent fibrin degradation, which are characteristics of sepsis-induced coagulopathy.

During sepsis, the anticoagulant and fibrinolytic functions of endothelial cells are compromised, leading to platelet aggregation and coagulation cascade activation (Fig. 1). The anticoagulant functions of endothelial cells are impaired, mainly manifested as increased expression of TF on the surface of activated endothelial cells. Phosphatidylserine (PS) is exposed on the outer membranes of damaged endothelial cells, providing a high-affinity binding site for coagulation factors. Additionally, hemodilution decreases the concentrations of coagulation factors and plasma anticoagulant proteins, leading to uncontrolled clotting on the surface of activated endothelial cells [43]. Additionally, activated endothelial cells express TFs and secrete PS-positive microparticles, which amplify local and disseminated coagulation [49]. Extracellular histones interact with prothrombin, promoting FXa-mediated prothrombin cleavage and the release of active thrombin [50]. The extrinsic coagulation pathway, which is dependent on TF from blood cells, also contributes to intravascular coagulation, which primarily occurs on microvesicles derived from activated monocytes and the surface of activated endothelial cells [51]. During sepsis, the levels of plasminogen activator inhibitor-1 (PAI-1) significantly increase, resulting in fibrinolytic system shutdown and further exacerbation of microvascular thrombosis [52]. In mice infected with *Escherichia coli* or *S. aureus* or injected with LPS, substantial thrombin generation can be observed in the hepatic microcirculation [53]. Consistent with this, most patients with sepsis exhibit abnormalities in coagulation and fibrinolysis upon admission, with elevated levels of thrombin–antithrombin (TAT; a complex thrombin generation marker) in 98.7% of patients, increased levels of fibrin degradation products (FDPs) in 97.4% of patients, and decreased protein C activity in 88.3% of patients [54].

Activated leukocytes significantly contribute to the development of intravascular coagulation and the subsequent cytokine storm. Under microbial stimulation, activated neutrophils secrete NETs, which provide a framework for and trigger intravascular coagulation [55]. NETs consist of neutrophil serine proteases, DNA, and histones, all of which contribute to the activation of coagulation. The negatively charged DNA surface triggers FXII-dependent coagulation by binding and activating FXII, which acts as an initiator of the intrinsic coagulation pathway [56]. Neutrophil serine proteases can deactivate TFPI (an anticoagulant protein) [57]. Sepsis-associated coagulopathy and the related impairment of microvascular blood flow are also caused by the interaction between platelets and endothelial cells [58]. In sepsis, platelets create platelet–leukocyte aggregates and facilitate neutrophil transendothelial migration to restrict bacterial spread. These aggregates eventually attach to the endothelium, exacerbating local inflammation and leading to further tissue hypoperfusion. In thrombin-activated platelets, the up-regulation of CD40-L and GPIIbIIIa expression can stimulate chemokine secretion, up-regulate adhesion molecule expression, and increase TF release by endothelial cells, exacerbating microvascular thrombosis in the context of sepsis [59].

## Interaction of Endothelial Cells with Other Cells in the Immunoinflammatory Response

**Fig. 2.**
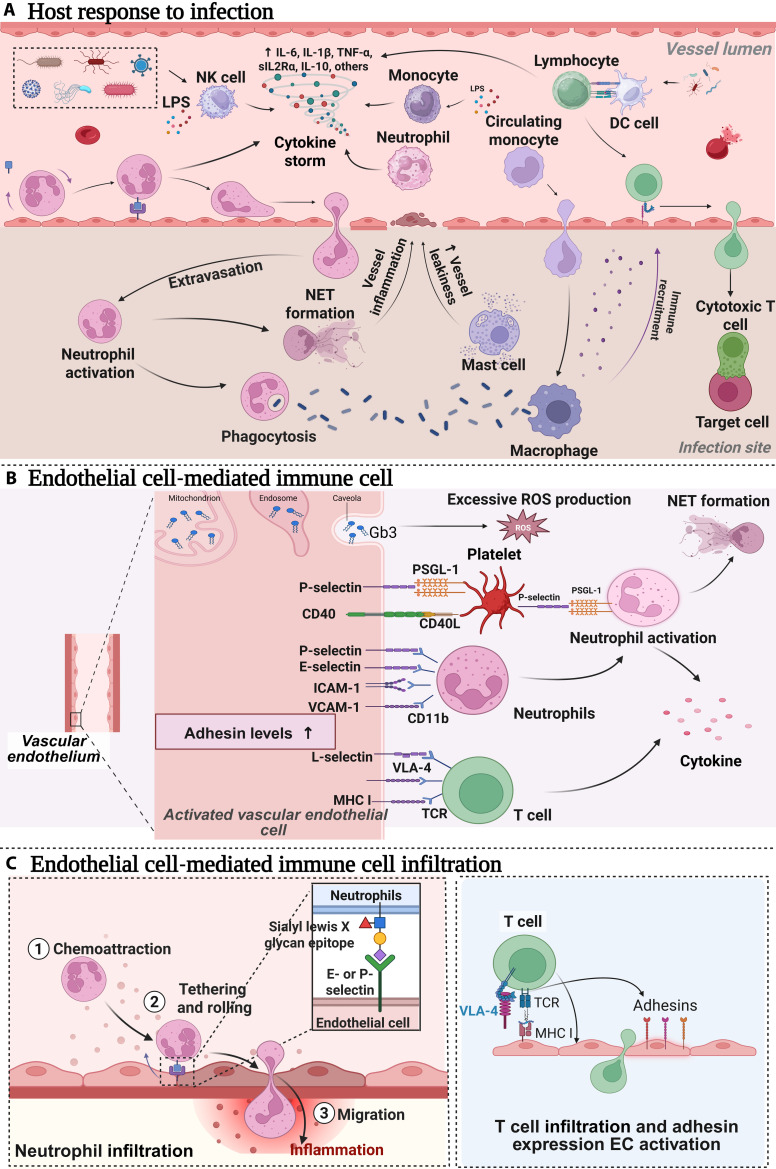
Interactions between endothelial cells and immune cells in sepsis and the pathophysiological processes mediating immune cell infiltration. (A) Host response to infection: During infection, pathogenic microorganisms activate natural killer (NK) cells and neutrophils, triggering a cytokine storm that releases cytokines such as IL-6, IL-1B, TNF-α, sIL-2Rα, and IL-10. Monocytes and circulating monocytes are activated and differentiate into macrophages, while lymphocytes and DCs also participate in the immune response. Neutrophils contribute to immune defense through the formation of neutrophil extracellular traps (NETs) and phagocytosis. (B) Endothelial cell-mediated immune cell adhesion: Endothelial cells promote the adhesion and migration of immune cells, such as neutrophils and T cells, by increasing the expression of adhesion molecules (such as P-selectin, E-selectin, ICAM-1, VCAM-1, and CD11b). Endothelial cells can also bind to platelets through P-selectin and CD40, and platelets can bind to neutrophils through P-selectin/PSGL-1, resulting in endothelial cell–platelet–leukocyte interactions. Additionally, endothelial cells participate in the immune response through the release of microparticles and the accumulation of Gb3. (C) Endothelial cell-mediated immune cell infiltration: Activated vascular endothelial cells facilitate the infiltration of neutrophils and T cells by increasing the expression of adhesion molecules [such as E- or P-selectin and major histocompatibility complex class I (MHC I)]. Neutrophils roll, adhere, and migrate across the vascular wall to enter the site of inflammation. T cells interact with VLA-4 and MHC I, express adhesion molecules, and become activated to participate in the immune response.

During the development of sepsis, the interaction between endothelial cells and immune cells plays a crucial role (Fig. 2). Endothelial cells are the main components of the vascular wall and are important immune regulatory cells that can sense pathogen signals and participate in immune responses. The activation of endothelial cells is a crucial initial step in the inflammatory response during sepsis. When pathogens or their products (such as LPSs) enter the bloodstream, they can activate endothelial cells through TLRs, leading to an increase in the transcription of proinflammatory genes in endothelial cells. Activated endothelial cells undergo significant morphological and functional changes, primarily characterized by cellular contraction, gap formation, and alterations in the expression of surface molecules [60]. When activated, proinflammatory endothelial cells release various proinflammatory cytokines, such as IL-6, CCL2, and INF-α, which participate in the immune response [61]. Moreover, immune cells such as monocytes, neutrophils, and macrophages are activated after the recognition of PAMPs and DAMPs through TLRs and Fcγ receptors. These activated immune cells also release many proinflammatory cytokines (such as IL-1β, IL-6, and TNF-α) and chemokines that participate in the immune response [62].

### The interaction between endothelial cells and immune cells

Endothelial cells are instrumental in the recruitment and migration of immune cells. Activated endothelial cells present several adhesion molecules on their surface, such as vascular cell adhesion molecule-1 (VCAM-1), ICAM-1, and platelet endothelial cell adhesion molecule-1 (PECAM-1). These molecules can bind to integrins (such as LFA-1 and VLA-4) on the surface of immune cells, promoting the adhesion and migration of immune cells on the endothelial surface [63]. Activated endothelial cells also secrete a variety of chemokines, such as CXCL8 (IL-8) and CCL2 (MCP-1). These chemokines form concentration gradients within blood vessels, guiding immune cells such as neutrophils and monocytes to migrate toward the site of infection [64]. Endothelial dysfunction also promotes the recruitment of monocytes and amplifies inflammatory signaling, thereby exacerbating vascular damage [65].

In addition, the interaction between endothelial cells and immune cells not only promotes the recruitment and migration of immune cells but also contributes to their activation. Neutrophils are the primary effector cells involved in the early response to sepsis and kill pathogens by releasing ROS and proteases. However, the overactivation of neutrophils can lead to tissue damage. Endothelial cells promote the activation and function of neutrophils through the release of chemokines such as IL-8 and the expression of adhesion molecules such as ICAM-1 [66].

Monocytes and macrophages also play significant roles in the pathogenesis of sepsis. Endothelial cells can recruit monocytes by releasing chemokines such as MCP-1 and promote the differentiation of monocytes into macrophages through cell-to-cell contact and cytokine secretion. Activated macrophages can release substantial amounts of proinflammatory cytokines, such as IL-1β and TNF-α, further amplifying the inflammatory response. Additionally, endothelial cells can regulate T cell function through the expression of molecules such as PD-L1, which is involved in the regulation of adaptive immune responses [67,68].

The interaction between immune cells and endothelial cells forms a positive feedback loop that further amplifies the inflammatory response. Cytokines and chemokines released by endothelial cells activate more immune cells, while the activation of immune cells further damages endothelial cells. This vicious cycle leads to the persistence of systemic inflammatory response syndrome (SIRS). Additionally, in the late stages of sepsis, patients often develop immunosuppression, which is closely associated with the interactions between endothelial and immune cells. Persistent endothelial cell activation leads to increased expression of immunosuppressive molecules, such as PD-L1 and indoleamine 2,3-dioxygenase (IDO). These molecules can inhibit T cell activation and function, leading to immunosuppression [69]. During sepsis, the rate of apoptosis in endothelial cells and immune cells (particularly lymphocytes) significantly increases. This excessive apoptosis leads to a reduction in immune cell numbers and impaired function, resulting in an immunosuppressive state. Endothelial cells can induce immune cell apoptosis through the Fas/FasL pathway and secrete soluble factors, whereas immune cells can induce endothelial cell apoptosis through the release of cytotoxic substances [70].

### Interactions among endothelial cells, platelets, and leukocytes

Endothelial cell–platelet–leukocyte interactions play crucial roles in the migration of immune cells and the enhancement of immune responses during sepsis. Platelets are versatile cells that play key roles in hemostasis, thrombosis, immune responses, and angiogenesis, among other physiological and pathological processes [71,72]. In addition, platelets act as important inflammatory effector cells capable of influencing both innate and adaptive immune responses [73–78]. During sepsis, inflammatory cytokines stimulate endothelial cells to express P-selectin, which binds with P-selectin glycoprotein ligand-1 (PSGL-1) on the surface of platelets, mediating the initial adhesion of platelets to endothelial cells. To achieve more stable adhesion, platelets can bind with endothelial cells further through surface integrins (such as GPIIb/IIIa) [79]. Activated platelets secrete inflammatory cytokines (such as RANTES and IL-1) and chemokines, which further activate endothelial cells and leukocytes, forming a vicious cycle of inflammation and thrombosis. Platelets bind CD40 on the surface of endothelial cells through secreted CD40L (CD154), promoting the secretion of chemokines by endothelial cells and the expression of adhesion molecules and enhancing the inflammatory response [27]. In addition, activated platelets bind to PSGL-1 on leukocytes through the expression of P-selectin [80]. Fibrinogens on platelets can also interact with integrins on leukocytes. These interactions enable signal transduction between platelets and leukocytes [81,82]. Through platelet–leukocyte interactions, platelets provide leukocytes with appropriate inflammatory signals, inducing them to transcribe IL-1β and NLRP3 and enhancing their response to canonical inflammasome triggers [such as adenosine triphosphate (ATP) and nigericin], thereby exacerbating the inflammatory response [83]. In addition, during sepsis, platelet–leukocyte complexes are important factors in the disruption of the microcirculation. Platelets coordinate pathological immune reactions and promote the recruitment of leukocytes to target tissues. Platelets, neutrophils, and monocytes are activated through TLR4 ligand binding, which induces the binding of platelets to leukocytes, ultimately leading to the strong activation of neutrophils in the form of NETs [83]. Despite the aforementioned detrimental effects of overactivated NETs, such as damage to endothelial cell membranes, disruption of the endothelial barrier, and the promotion of immunothrombosis, these structures play irreplaceable roles in the host [84]. Through a physical “web-trapping” mechanism, NETs immobilize pathogenic microbes and prevent their dissemination, while the antimicrobial proteins embedded on them (e.g., cathepsin G, myeloperoxidase, and defensins) directly kill the pathogens. Moreover, NETs polarize macrophages toward the M1 phenotype, increasing their bactericidal capacity; induce dendritic cell maturation to promote antigen presentation; stimulate B cells to secrete B cell activating factor (BAFF), thereby bridging innate and adaptive immunity; and, via gasdermin-D pores, release IL-1β to recruit additional neutrophils to the site of infection [85,86].

The complement system also plays a significant role in the interaction between endothelial cells and immune cells. Complement system activation leads to the recruitment and activation of immune cells such as neutrophils and macrophages, thereby exacerbating endothelial injury [87]. In addition, the interaction between the complement system and immune cells can amplify the inflammatory response, thereby contributing to the persistence of systemic inflammation in sepsis [88]. Endothelial cells interact with immune cells via these pathways, leading to increased vascular permeability and microcirculatory disturbances, which affect organ perfusion and oxygenation. This pathophysiological process ultimately results in MODS, which is a major cause of death in patients with sepsis [89].

## Role of Endothelial Cell and Barrier Function Protection in the Treatment of Sepsis: Potential Therapeutic Approaches

Considering the crucial role of endothelial cells in sepsis, therapeutic strategies aimed at protecting endothelial cells hold great potential. Restoring endothelial barrier function and mitigating inflammatory and coagulopathy-related damage are essential for improving the prognosis of patients with sepsis (Tables 1 and 2) [90].

**Table 1.** Medicinal interventions targeting endothelial dysfunction in sepsis

Target	Drug	Animal research (conclusions)	Clinical research (cohort conclusions)	Reference
Protecting the glycocalyx	Human serum albumin (HSA)	Protects the endothelial glycocalyx, maintains plasma colloid osmotic pressure, scavenges free radicals, and regulates immune function.	Hypertonic albumin solutions can improve the outcomes of patients with septic shock	[63,91,92]
Protect the glycocalyx	Hydrocortisone	Prevents TNF-α-induced shedding of the endothelial glycocalyx.		[93]
Inflammatory factors IL-6 and TNF-α	Recombinant human thrombopoietin (rhTPO)		Reduces the levels of inflammatory factors and thereby alleviates sepsis-induced endothelial cell damage	[95]
eNOS cofactors	Tetrahydrobiopterin (BH4)	Restores endothelial function, increases microcirculatory perfusion ratios and functional capillary density		[96]
Histone deacetylase 6 (HDAC6)	Tubastatin A (TubA)	TubA is a selective HDAC6 inhibitor that specifically targets the deacetylase activity of HDAC6. It prolongs survival in septic mice by inhibiting the death of pulmonary vascular endothelial cells, alleviating inflammatory responses, and restoring endothelial barrier function.		[99]
Phosphodiesterase 4 (PDE4)	Phosphodiesterase 4 inhibitor	Prevents the disruption of endothelial barrier structure		[100]
Tie2 receptor	Angiopoietin-1 (ANG1)	It binds to the Tie2 receptor on the surface of endothelial cells to exert its biological functions. It enhances the connections between endothelial cells, has anti-inflammatory effects, and promotes the survival and repair of endothelial cells.		[105]
Angiopoietin–Tie pathway	Matrix metalloproteinase 14 (MMP14) inhibitor	By inhibiting MMP14, it promotes vascular remodeling and repair, suppresses inflammatory responses, and enhances the survival and anti-apoptotic properties of endothelial cells.		[103]
Angiopoietin-2	Bifonazole (BIFO)	Reduces the biosynthesis and release of Angpt-2 by endothelial cells under both basal and inflammatory conditions, protects endothelial cells, improves vascular barrier function, and reduces vascular leakage.		[104]
Adrenomedullin (ADM)	Adrecizumab (ADZ) (a monoclonal antibody targeting ADM)	During sepsis, an increase in the concentration of ADM can lead to hypotension and vasodilation.	Clinical trials are currently underway to assess its safety and efficacy in patients with sepsis. ADZ demonstrated good safety in phase I studies in healthy subjects. In the first-in-human phase II study, biological therapy with ADZ was beneficial.	[107,108]
JAK/STAT3 and PI3K/AKT signaling pathways	JAK inhibitor and PI3K inhibitor	Inhibits the production of MCP-1 in endothelial cells, thereby suppressing inflammatory responses.		[110]
Tripartite motif containing 47 (TRIM47)	TRIM47 inhibitor	Inhibiting the expression of TRIM47 can suppress the transcription of various proinflammatory cytokines in endothelial cells, reduce the adhesion of monocytes and the expression of adhesion molecules, and inhibit the secretion of IL-1β and IL-6 by endothelial cells, thereby alleviating endothelial inflammation.		[111]
ATP-citrate lyase (ACLY)	ATP-citrate lyase (ACLY) inhibitor	Inhibition of ACLY can significantly alleviate LPS-induced inflammatory responses in endothelial cells, reducing the expression of adhesion molecules and the production of MCP-1 in these cells.		[112]
Nuclear factor κ-light-chain-enhancer of activated B cells (NF-κB)	Statins	Effectively inhibits TNF-α-induced NF-κB activity in endothelial cells stimulated by proinflammatory stimuli.		[113]
Thrombin	Recombinant human soluble thrombomodulin (rhsTM)	rTM is an anticoagulant protein similar to endogenous thrombomodulin. It binds to thrombin to produce activated protein C (APC), which can limit the amplification of coagulation without prolonging clotting time.	rhsTM reduces the 28-d mortality rate in patients with sepsis-associated coagulopathy (SAC), but it is ineffective in sepsis patients without SAC.	[114]
Activated coagulation factors	Antithrombin γ (rAT)	rAT is a recombinant alternative to human plasma-derived antithrombin. It can capture activated coagulation factors, including thrombin and FXa, to achieve anticoagulation.	Recombinant antithrombin (rAT) has similar safety and efficacy to plasma-derived antithrombin (pAT) in treating sepsis-induced disseminated intravascular coagulation (DIC). Clinical results support the use of rAT as an alternative to pAT to improve the prognosis of patients with DIC.	[115,116]
Protease-activated receptor (PAR)	APC	Limits the increase in endothelial permeability and restricts the amplification of coagulation by influencing PAR.		[117,118]
Ultra-large von Willebrand factor (ULVWF) multimers	ADAMTS13	It can cleave ULVWF (ultra-large von Willebrand factor) into smaller VWF (von Willebrand factor) molecules, thereby reducing thrombus formation in vascular injury.		[121,122]
	Endothelial progenitor cells (EPCs)	Primarily secrete proangiogenic cytokines. Late-stage EPCs (endothelial progenitor cells) have a higher proliferative potential and ability to form blood vessels.		[126]
	Mesenchymal stromal cells (MSCs)	MSCs primarily increase the expression of VE-cadherin on the cell membrane through the production of secretory factors after interacting with endothelial cells.		[128]
Tyrosine kinase	Imatinib	Imatinib protects microcirculatory perfusion and oxygenation by alleviating inflammation and endothelial activation, thereby decreasing organ damage.	Imatinib protects microcirculatory perfusion and oxygenation during and after cardiopulmonary bypass (CPB) by reducing endothelial barrier dysfunction and vascular leakage.	[131–133]
Proprotein convertase subtilisin/kexin type 9 (PCSK-9)	PCSK-9 inhibitors	In PSCK-9-deficient mice, decreased expression of NADPH oxidase and reduced ROS (reactive oxygen species) production were observed, which protected the endothelial barrier.		[136–139]
Rab11-dependent pathway	Intermedin (IMD)	IMD can increase the expression of VE-cadherin in vascular endothelial cells through a Rab11-dependent pathway, repair damaged endothelial cell junctions, and reduce vascular leakage. IMD can also reduce inflammatory responses in septic mice, lowering the levels of inflammatory cytokines such as TNF-α, IL-1β, IL-6, and MCP-1 in the serum.		[140,141]
Therapeutic plasma exchange			TPE can partially alleviate the imbalance in the coagulation system in sepsis patients and protect endothelial cells.	[142–145]
C5a	Anti-C5a monoclonal antibody (vilobelimab)	Primarily inhibits neutrophil chemotaxis and endothelial activation by blocking the binding of C5a to its receptor C5aR1.	The relevant clinical trial has been completed, but the results have not yet been publicly released	[147–149]
C1	C1-esterase inhibitor (C1-INH)	Early inhibition of the complement system, particularly regarding its effects on neutrophil function.	The relevant clinical trial has been completed, but the results have not yet been publicly released	[146,149,151]
C3	C3 inhibitor (Cp40)	Protects the endothelial barrier and reduces microthrombus formation by inhibiting the onset of systemic inflammation, thereby markedly attenuating organ injury in *E. coli*-induced sepsis.		[152,153]

**Table 2.** Development stage and principal targets of therapeutic strategies for sepsis-induced endothelial dysfunction

Therapeutic class	Principal target/mechanism	Clinical stage	Representative agent/approach	References
Glycocalyx protectants	Stabilizes the glycocalyx; combats oxidative stress	Preclinical/early phase	Hyperosmotic albumin (HAS)	[91,92]
Anti-inflammatory small molecules	HDAC6, PDE4, ACLY, and TRIM47	Preclinical/early phase	Tubastatin A and PDE4 inhibitors	[99,100,111,112]
Endothelial barrier stabilizers	Tie2, VE-cadherin, and Angpt-1/2	Preclinical/early phase	Angiopoietin-1, bifonazole, and MMP14 inhibitors	[103–105]
Endothelial barrier stabilizers	Adrenomedullin (ADM)	Phase II	Adrecizumab (ADZ)	[107,108]
Anticoagulant agents	Coagulation cascade (TF, APC, and ULVWF)	Phase III	rhTM, rAT, ADAMTS13, and N-acetylcysteine	[114–116,121,122]
Complement-targeted therapy	C3, C5, C5a, and C1-INH	Phase II–III	Vilobelimab, Cp40, and C1-esterase inhibitor	[146–153]
Stem/cell-based therapy	Endothelial repair; paracrine effects	Preclinical/early phase	MSCs and EPCs	[126,128]
Tyrosine kinase inhibitors	Arg/Abl2 and PDGFR	Phase II (COVID-19)	Imatinib	[131–133]
PCSK9 inhibitors	TLR4/NF-κB/NLRP3 axis	Phase II (COVID-19)	Evolocumab	[136–139]
Peptide barrier protectants	CLR/RAMP2 receptor	Preclinical	Intermedin (IMD)	[140,141]
Plasma-based therapy	Replaces deficient proteins; removes inflammatory mediators	Retrospective clinical studies	Fresh frozen plasma (FFP) and therapeutic plasma exchange	[142–145]

### Protecting endothelial cells and endothelial barrier function is a key therapeutic strategy for sepsis

During sepsis, shedding of the EG leads to increased vascular permeability and impaired microcirculation. Studies have shown that the tight binding of human ALB to the EG maintains colloid osmotic pressure, scavenges free radicals, and modulates immune function, protecting the EG [63]. Currently, no clinical studies have shown that hypertonic albumin solutions (HASs) can significantly reduce the overall mortality rate of patients with sepsis. However, HASs may be beneficial for the survival of patients with septic shock [91]. According to the Clinical Practice Guidelines for the Management of Sepsis and Septic Shock released by Japan in 2024, the use of HASs can improve the prognosis of patients with septic shock [92]. Additionally, hydrocortisone and antithrombin have been reported to prevent the TNF-α-induced shedding of the EG [93]. Some studies have demonstrated that endothelial cell protectants, such as recombinant human thrombomodulin (rhTM) and VEGF, can reduce endothelial cell injury, improve vascular barrier function, and mitigate organ damage [94]. The results of a retrospective clinical study revealed that recombinant human thrombopoietin (rhTPO) can reduce the levels of inflammatory factors and alleviate sepsis-induced endothelial cell damage by activating the phosphatidylinositol 3-kinase (PI3K)/AKT pathway [95]. During sepsis, endothelial nitric oxide synthase (eNOS) dysfunction leads to reduced vascular tone and excessive ROS production. Tetrahydrobiopterin (BH4), a key cofactor of eNOS, plays a crucial role in maintaining vascular tone by regulating eNOS function under physiological conditions. Studies have shown that BH4 has the capacity to restore endothelial function, enhance microcirculatory flow, and increase functional capillary density [31,96]. Recent research has indicated that specific knockdown of the CLEC5A gene in endothelial cells can exert a protective effect on the endothelial barrier [97]. In septic mice, prophylactic or therapeutic administration of EphA4-Fc, a decoy receptor and pan-ephrin inhibitor, can increase survival rates and reduce vascular leakage, lung injury, and endothelial cell dysfunction. Additionally, in vitro experiments have confirmed that the administration of EphA4-Fc to cultured human endothelial cells can prevent the loss of endothelial junction proteins, specifically VE-cadherin, after TNF-α pretreatment, thereby maintaining the integrity of the endothelial cell barrier [98].

Histone deacetylase 6 (HDAC6) is widely expressed in endothelial cells, where it regulates late-stage histone deacetylation and affects endothelial barrier permeability. By modulating the transcription of proinflammatory genes and strengthening intercellular junctions, HDAC6 inhibitors can protect the endothelial barrier [99]. Wu et al. [99] demonstrated that TubA, a highly selective HDAC6 inhibitor, can reduce the mortality of septic mice by reducing pulmonary vascular endothelial cell death, suppressing inflammation, and protecting endothelial barrier function. Additionally, the systemic administration of phosphodiesterase 4 (PDE4) inhibitors may help stabilize the microvascular barrier and improve microcirculatory blood flow in patients with sepsis [100].

Targeting the angiopoietin–Tie pathway is also a therapeutic approach to maintain vascular endothelial barrier function. Angiopoietin-1 (ANG1) acts as a strong agonist of TIE2, protecting endothelial cells and maintaining the integrity of endothelial barrier function. In contrast, angiopoietin-2 (ANG2) functions as a weak agonist or antagonist of TIE2, and its effects depend on the cellular microenvironment [101]. Under inflammatory conditions, increased ANG2 expression leads to vascular leakage and inflammatory responses [102]. Recent studies have shown that matrix metalloproteinase-14 (MMP14) cleaves the fibronectin type III (FN3) domain of Tie2, leading to decreased Tie2 expression. This enzyme is considered the primary cause of Tie2 down-regulation. Inhibiting MMP14 reduces Tie2 cleavage, alleviating sepsis-induced vascular leakage and inflammatory responses [103]. Pape et al. [104] demonstrated that bifonazole (BIFO) reduces the biosynthesis and release of Angpt-2 by endothelial cells under both basal and inflammatory conditions, improving vascular barrier function and reducing vascular leakage. ANG1 restores intercellular junctions in endothelial cells by activating Rac1 and inhibiting RhoA, reducing the formation of cell gaps and improving vascular barrier function [105].

Adrenomedullin (ADM)-targeted therapies have also emerged as potential treatments for protecting the vascular endothelial barrier in sepsis treatment. ADM is a freely circulating peptide that is expressed and secreted primarily by vascular endothelial cells. During sepsis, increased ADM concentrations lead to hypotension and vasodilation [106]. As a humanized monoclonal antibody, adrecizumab (ADZ) targets the N terminus of ADM and is designed to inhibit excess circulating ADM in the setting of sepsis and stimulate the protective effects of the endothelial barrier while reducing interstitial vasodilation. ADZ is the first humanized monoclonal antibody that targets bioactive ADM. By binding to excess circulating ADM in patients with sepsis, ADZ enhances the endothelial barrier-protective effects of ADM and reduces interstitial vasodilation [107]. In phase I studies involving healthy individuals, ADZ demonstrated good safety. Additionally, in the first-in-human phase II study, ADZ demonstrated some therapeutic benefits [107]. In a phase II multicenter clinical trial named AdrenOSS-2 (clinical trial identification number NCT02338843), ADZ treatment was associated with a reduction in 28-d mortality in the overall study cohort, but the difference was not statistically significant [hazard ratio (HR), 0.84; 95% confidence interval (CI), 0.53 to 1.31; *P* = 0.439]. However, in the subgroup of patients with low levels (<50 ng/ml) of circulating dipeptidyl peptidase 3 (cDPP3), the therapeutic effect of ADZ was more pronounced (HR, 0.61; 95% CI, 0.34 to 1.08; *P* = 0.085) [108]. Further research is needed to confirm the safety and clinical efficacy of ADZ in the treatment of sepsis.

### Modulating endothelial cell-mediated inflammatory responses is another important therapeutic strategy

The development of specific inhibitors that target proinflammatory cytokines and adhesion molecules released by endothelial cells may help control excessive inflammatory responses. For example, anti-TNF-α antibodies and ICAM-1 inhibitors have shown efficacy in clinical trials [109]. According to Zegeye et al. [110], IL-6 trans-signaling activates the Janus kinase (JAK)/signal transducer and activator of transcription 3 (STAT3) and PI3K/AKT pathways, inducing proinflammatory responses in human vascular endothelial cells. Pretreatment with JAK or PI3K inhibitors can inhibit MCP-1 production in endothelial cells, thus suppressing the inflammatory response. TRIM47, an E3 ubiquitin ligase, is highly expressed in vascular endothelial cells and acts as a novel endothelial activation factor; it exacerbates LPS-induced acute lung injury (ALI) by enhancing K63-linked TRAF2 ubiquitination. By inhibiting the expression of TRIM47, the transcription of various proinflammatory cytokines in endothelial cells can be reduced, along with decreased monocyte adhesion and expression of related adhesion molecules, alleviating the inflammatory response of endothelial cells [111]. ATP-citrate lyase (ACLY) promotes inflammatory responses and glycolipid metabolism in endothelial cells (ECs) by increasing the acetylation of FoxO1 and histone H3 and increasing MYC transcription. Inhibiting ACLY significantly alleviates LPS-induced endothelial inflammation, reducing the expression of endothelial adhesion molecules and MCP-1 [112]. Statins reduce endothelial inflammation by effectively inhibiting TNF-α-induced NF-κB activity in endothelial cells stimulated by proinflammatory factors [113].

### Improving endothelial-related coagulopathy is an important therapeutic direction for sepsis

During sepsis, the disruption of the EG, down-regulation of endothelial thrombomodulin, and decreased expression of plasma anticoagulant proteins are the main reasons for the compromised anticoagulant potential in the vascular environment. Studies have shown that recombinant thrombomodulin (rTM) and recombinant antithrombin γ (rAT) are promising anticoagulant agents for the treatment of sepsis-associated microcirculatory coagulopathy. rTM is an anticoagulant protein that generates activated protein C (APC) by binding to thrombin, limiting coagulation amplification without prolonging the clotting time [43]. Recombinant human soluble thrombomodulin (rhsTM) has clinical value in the treatment of patients with sepsis complicated by sepsis-associated coagulopathy (SAC). A phase III randomized clinical study (SCARLET) indicated that rhsTM could reduce 28-d mortality in patients with sepsis and SAC but that it was ineffective in those without SAC [114]. rAT, as an alternative to human plasma-derived antithrombin, can exert anticoagulant effects by capturing activated thrombin and FXa. Endo and colleagues [115,116] reported that rAT is as effective and safe as plasma-derived antithrombin (pAT) in treating sepsis-induced disseminated intravascular coagulation (DIC), indicating that rAT is a good alternative to pAT. APC primarily protects the endothelium by influencing protease-activated receptors (PARs), limiting endothelial permeability, and restricting the amplification of coagulation [117,118]. The 2-path unifying theory suggests that hemostasis can be activated through 2 independent pathways: the ultra-large von Willebrand factor (ULVWF) multimer pathway and the TF pathway. These pathways correspond to microthrombus formation and macrothrombus formation, respectively [119]. Traditional anticoagulant approaches targeting the TF pathway are limited in their ability to treat sepsis-associated microthrombus formation, as they cannot inhibit ULVWF multimer production [120]. ADAMTS13, a metalloproteinase that can reduce thrombosis caused by vascular injury, functions primarily by cleaving ULVWF multimers into smaller VWF fragments. Recombinant human ADAMTS13 can inhibit microthrombus formation via the ULVWF multimer pathway. N-acetylcysteine (NAC), an ADAMTS13 mimetic, has been shown to reduce diffuse intravascular microthrombus formation by inhibiting the ULVWF pathway [121,122]. It would be interesting to investigate whether VWF receptor inhibitors [123–125] can treat sepsis, as this is currently unknown.

### Other potential therapeutic approaches

#### Endothelial progenitor cells and mesenchymal stromal cells

Emerging therapies, such as endothelial progenitor cell (EPC) therapy and stem cell transplantation, may help accelerate endothelial repair and improve vascular function. EPCs are key cells involved in vascular repair, with early EPCs primarily secreting proangiogenic cytokines and late EPCs having greater proliferative potential and vascular formation capabilities [126]. Currently, EPCs and EPC-derived extracellular vesicles (EVs) have been used in therapeutic research for various diseases, but they have not yet entered the clinical research phase. Mesenchymal stromal cells (MSCs) can protect endothelial cells from barrier disruption induced by LPS [127]. MSCs promote the expression of VE-cadherin in endothelial cells and increase its binding to β-catenin through the secretion of paracrine factors [128]. Pati and colleagues [129] demonstrated that bone marrow (BM)-MSCs can protect the integrity of the pulmonary vascular endothelium and reduce the occurrence of pulmonary edema in a hemorrhagic shock model by inhibiting inflammation.

#### Imatinib and other tyrosine kinase inhibitors

Arg/Abl2 is a tyrosine kinase that has been identified as a mediator of endothelial barrier disruption [130]. The vascular barrier-protective effects of imatinib, the most extensively studied tyrosine kinase inhibitor, have been confirmed in various research models. Additionally, imatinib can mitigate oxidative damage, apoptosis, and pulmonary edema by increasing the activity of antioxidant enzymes in lung endothelial cells, and it reduces systemic inflammatory cytokine expression [131]. During the treatment of patients with COVID-19, many clinical phenomena indicated vascular barrier protection following imatinib treatment [132]. Another clinical study revealed that imatinib can significantly reduce the mortality rate of patients with severe COVID-19 and improve their oxygenation status, primarily by modulating innate immune responses and reversing endothelial dysfunction [133]. Therefore, increasing evidence supports the potential role of short-term imatinib administration as a therapeutic agent in maintaining endothelial barrier integrity and mitigating sepsis-associated inflammation.

#### PCSK-9 inhibitors

PCSK-9 levels are elevated in animal models of sepsis as well as in patients with sepsis [134]. Studies have shown that PCSK9 can increase inflammatory responses through the TLR4/NF-κB and NLRP3 pathways, leading to endothelial dysfunction in sepsis models. PCSK-9 inhibitors (PCSK-9i) can protect the endothelial barrier by reducing the impairment of VE-cadherin expression [134]. Zhang et al. [135] reported reduced NADPH (reduced form of nicotinamide adenine dinucleotide phosphate) oxidase expression in PCSK-9-deficient mice. NADPH oxidase is a major source of ROS, which may provide a new theoretical basis for developing strategies to protect the endothelium. Moreover, PCSK-9i exert anti-inflammatory effects by interfering with the NF-κB pathway to reduce the production of inflammatory cytokines [136]. Currently, 2 U.S. Food and Drug Administration (FDA)-approved PCSK-9is, evolocumab (Repatha) and alirocumab (Praluent), are primarily used for the treatment of hypercholesterolemia [137]. In a multicenter clinical trial, 60 patients with severe COVID-19 infection were randomly divided into a placebo group and an evolocumab treatment group. Compared with the placebo, the PCSK-9i reduced serum IL-6 levels in patients with severe COVID-19, decreased the duration of invasive respiratory support, and lowered the mortality rate of patients [138]. Another clinical study involving 25 patients with familial hypercholesterolemia (FH) added a PCSK-9i (Evolocumab 140 mg subcutaneous injection every 14 d) to their existing lipid-lowering therapy. After 12 weeks of treatment, low-density lipoprotein cholesterol (LDL-C) levels in patients were significantly decreased. These findings indicate that the observed results are associated with the significant improvement in endothelial function caused by PCSK-9is [139].

#### Intermedin

Intermedin (IMD), a member of the calcitonin family of peptides, has potential roles in maintaining endothelial barrier function and exerting anti-inflammatory effects [140]. IMD can promote the relocalization of VE-cadherin in endothelial cells via a Rab11-dependent pathway, repairing damaged endothelial junctions and reducing vascular leakage in the lungs, kidneys, and livers of septic mice. Additionally, IMD can exert anti-inflammatory effects by reducing the levels of inflammatory cytokines in the serum of septic mice [140]. Yang et al. [141] reported that IMD stabilizes the endothelial barrier through a specific receptor complex (CLR/RAMP2), suggesting that IMD may serve as a potential therapeutic agent for inflammation-related vascular leakage.

#### Plasma and therapeutic plasma exchange

Plasma contains various bioactive components, such as S1P and antithrombin, which may protect and restore the glycocalyx to maintain or restore vascular homeostasis. In particular, the administration of fresh frozen plasma (FFP) has demonstrated clinical potential in the treatment of trauma and sepsis [12]. In recent years, there have been increasing reports concerning the role of therapeutic plasma exchange (TPE) in protecting endothelial cells and improving endothelial barrier functioned. TPE removes inflammatory mediators, endotoxins, and other harmful factors from the plasma of patients with sepsis and replenishes FFP, restoring the normal function of endothelial cells [142]. After TPE, the degree of ultrastructural damage to endothelial cells is significantly reduced, and the tight junctions between cells are restored [143]. In clinical research, TPE has been shown to have better effects than heparin in patients with DIC, including increased platelet counts, improved coagulation function, and higher 28-d cumulative survival rates [144]. In a prospective single-center study of 31 patients with early-onset (within 12 h of onset) septic shock treated with high-dose norepinephrine (NE > 0.4 μg/kg/min), TPE significantly increased the activity of antithrombin III and protein C while reducing the levels of VWF antigen. These changes indicate that TPE can partially alleviate imbalances in the coagulation system in patients with sepsis, thereby protecting endothelial cells [145].

#### Targeted complement therapy

The development of drugs targeting the complement system has expanded from ultrarare disorders [e.g., paroxysmal nocturnal hemoglobinuria (PNH) and atypical hemolytic uremic syndrome (aHUS)] to common indications such as cancer, kidney diseases, and eye diseases. In sepsis, complement activation—via C3a, C5a, and MAC—drives endothelial cell activation, inflammation, and thrombosis, making it a central mechanism of organ injury. C5aR1 antagonists and C5 and C3 inhibitors have consistently been shown to protect the endothelium and improve organ function in preclinical models; however, clinical translation remains challenging [146].

##### Anti-C5a monoclonal antibody

A representative anti-C5a monoclonal antibody is vilobelimab (development codes IFX-1 or CaCP29), a humanized monoclonal antibody that blocks the binding of C5a to its receptor C5aR1, precisely targeting immune–endothelial crosstalk and thereby inhibiting neutrophil chemotaxis and endothelial activation [147,148]. A relevant clinical trial (registration number: NCT02246595) has been completed, but the results have not yet been publicly released [149]. Multiple authoritative reviews recognize it as the first systemic human trial of an anti-C5a monoclonal antibody for the treatment of sepsis; publication of the findings will be pivotal for validating the clinical value of complement inhibition in this setting. Moreover, vilobelimab has been shown to reduce mortality in patients with COVID-19-related acute respiratory distress syndrome (ARDS), indirectly supporting the potential of complement blockade to ameliorate sepsis-related endothelial injury and provide organ protection [150].

##### C1-esterase inhibitor (C1-INH)

A clinical trial (NCT01766414) is underway to evaluate the immunomodulatory effects of C1-INH in a human endotoxemia model, with a particular focus on neutrophil function [149]. The study highlights the potential role of C1-INH in early complement inhibition and represents a key investigation into the impact of complement blockade on early immune modulation in sepsis, potentially providing a theoretical basis for immunomodulatory therapies for this condition [146,151]. Recent information indicates that the trial has been completed, but the findings have not yet been made public.

##### C3 inhibitors

A representative C3 inhibitor is Cp40, a compstatin-derived C3 inhibitor that prevents C3 convertase-mediated cleavage of C3, thereby blocking the downstream generation of C3a, C5a, and the MAC [152]. In animal studies, Cp40 was shown to protect the endothelial barrier, reduce microthrombus formation, and markedly attenuate organ injury in *E. coli*-induced sepsis by suppressing the systemic inflammatory response [153].

##### Complement-targeted therapy combined with immune modulation

Combination strategies may become key approaches for enhancing the efficacy of complement-targeted interventions. Studies of animal models have shown that CD14 acts as a TLR4 coreceptor, participating in LPS recognition and amplifying inflammatory signals [154]. The simultaneous blockade of C3/C5 together with CD14 synergistically suppresses inflammatory cytokine release, markedly improves endothelial function and organ protection, and increases survival rates [155,156].

## Perspective for Future Research

As the vascular pioneers where infection, immunity, and coagulation first intersect, endothelial cells are no longer viewed as passive barriers but as organ-specific, dynamic orchestrators of sepsis pathophysiology. Future sepsis-focused research on endothelial cells should concentrate on the following topics.

The mechanisms regulating endothelial metabolic reprogramming and how glycolysis, fatty acid oxidation, and amino acid metabolism shape barrier integrity and immunomodulatory functions need to be elucidated, with a particular emphasis on the precise roles of metabolites such as succinate, lactate, and itaconate in regulating inflammatory signaling and determining endothelial cell fate. The dynamic feedback circuitry among the endothelium, immunity, and thrombosis should be further investigated by deeply dissecting the multidirectional regulatory network linking endothelial cells with immune cells (macrophages, T cells, and neutrophils) and platelets, with a special focus on the molecular switches that govern the immune-paralysis phase. Time series multiomics data and data on the temporal dynamics of endothelial phenotypic transitions should be leveraged to construct a stage-resolved atlas of endothelial evolution across sepsis. Single-cell sequencing and spatial transcriptomics should be used to dissect organ- and vascular bed-specific endothelial heterogeneity and functional zoning, thereby delineating sepsis-specific endothelial responses and revealing functional disparities in organ-specific injury patterns to define precise temporal windows and targets for intervention. Finally, the translational potential of endothelial repair and regeneration strategies should be integrated to evaluate, at both the mechanistic and clinical levels, the ability of EPCs, mesenchymal stem cells, and their EVs to promote vascular repair and restore barrier integrity.

Research on these topics will provide novel frameworks for understanding sepsis pathogenesis and guiding precision therapeutics, ultimately improving patient outcomes.

## Conclusion

Endothelial cells are the central hubs of the pathological process of sepsis. During sepsis, a subset of endothelial cells rapidly shifts to a procoagulant, proinflammatory phenotype, while the majority lose their glycocalyx and undergo multiple forms of cell death, disrupting endothelial barrier integrity. Their reciprocal activation with platelets and leukocytes amplifies localized infection into a systemic cascade. Both drive microvascular thrombosis and multiple organ failure. Conversely, restoring the endothelial barrier, such as by restoring heparan sulfate on the surface or blocking ADAM10/17, ADM, TRIM47 or PCSK-9 signaling, Ang-1, BH4, HDAC6 inhibitors, rhsTM, or ADAMTS13, or via TPE, restores barrier function. Whether adjunctive anti-platelet strategies can further rebalance coagulation and immunity and thereby improve survival warrants investigation in preclinical and early clinical studies. Future work must translate these mechanism-based interventions into more precise regimens with clearly defined optimal timing and combinations, transforming the microvasculature from a target of sepsis to a therapeutic gateway.
